# Current Status and Global Research Trend Patterns of Insect Meal in Aquaculture From Scientometric Perspective: (2013–2022)

**DOI:** 10.1155/2024/5466604

**Published:** 2024-10-22

**Authors:** Raghuvaran N., Tincy Varghese, Prasanta Jana, Angela Brighty R. J., Muthiah Sethupathy A., Sudarshan S., Yahya Bin Abdullah Alrashdi, Adel Ehab Ibrahim, Sami El Deeb

**Affiliations:** ^1^Fish Nutrition, Biochemistry and Physiology Division, ICAR—Central Institute of Fisheries Education, Mumbai 400061, Maharashtra, India; ^2^Department of Aquaculture, College of Fisheries Science, Birsa Agricultural University, Gumla, Ranchi 835207, Jharkhand, India; ^3^Fisheries Resource Harvest and Post-Harvest Management Division, ICAR—Central Institute of Fisheries Education (CIFE), Mumbai 400061, Maharashtra, India; ^4^Department of Aquatic Environment Management, TNJFU Dr. MGR Fisheries College and Research Institute, Thalainayeru 614712, Tamil Nadu, India; ^5^College of Pharmacy and Nursing, University of Nizwa, Birkat Al Mauz, Nizwa 616, Oman; ^6^Natural and Medical Sciences Research Center, University of Nizwa, P.O. Box 33, Birkat Al Mauz, Nizwa 616, Oman; ^7^Institute of Medicinal and Pharmaceutical Chemistry, Technische Universitaet Braunschweig, Braunschweig, Germany

**Keywords:** aquaculture, global research, insect meal, scientometric

## Abstract

In the past decade, insect meal has gained popularity in the animal feed industry, particularly in aquafeed, due to rising costs and decreased availability of fish meal (FM) and fish oil. Initially met with skepticism, insect meal is now seen as a promising ingredient because of its high nutrient profile. Research worldwide is exploring its potential as a FM replacement. Insects are abundant, nutritious, and environmentally friendly, as they can be reared on organic waste, minimizing the need for land, water, and energy. This research aims at obtaining a comprehensive and in-depth understanding of the current status and research trend patterns in this research field. To achieve this goal, this study conducts a mini systematic review and scientometric analysis of the global research published from 2013 to 2022 on the usage of insect meal in aquaculture. In the scientometric analysis, a total of 354 papers published by 1800 authors in 124 different journals from the Web of Science (WoS) core collection were analyzed, evaluating the number of publications, most relevant authors, organizations, top cited countries, most globally cited publications, and trending research themes in this field. The result showed that the University of Turin was the leading organization in insect meal research, whereas aquaculture was the leading journal, and author Laura Gasco was the prominent researcher in this field in the studied time frame (2013–2022). Italy was the leading country in Europe, while China dominated Asia in terms of the number of publications. The annual growth rate in insect meal research was found to be positive (23.11%), with 36.95 average citations per document. This study helps practitioners and scholars understand the current state of insect meal in aquaculture and identifies research requirements that can benefit both academia and industry.

## 1. Introduction

Aquaculture has been increasingly contributing to the food production as fish protein source where wild capture has more or less stagnated within the past decade [1, 2]. The increasing demand from aquaculture coupled with stagnation in wild catch has resulted in an increase in the cost of major feed ingredients such as fish meal (FM) and fish oil [3]. With increasing stocking densities in super intensive culture farms, feed has become the major contributor (50%–60%) of the capital cost in modern aquaculture practices. Thus, there is need to look out for sustainable alternative ingredients to replace FM without affecting the growth and health of the cultured species. Therefore, many research has been conducted in this regard during the last decade and many new ingredients have been tried to replace FM partially or completely [4].

Many plant ingredients (such as soyabean meal, rapeseed meal, cotton seed meal, and many oilseeds) have been tried, but the lower protein content and presence of antinutritional factors limited their usage in aquaculture. Several strategies have been also deployed to overcome these shortcomings. Usage of exogenous enzymes such as phytase and cellulase have been tried to reduce the antinutritional content in the plant ingredients. Strategies based on fermentation also tend to increase the protein content and reduce antinutritional factors, but still, they lack critical amino acids such as lysine and methionine which reduce their suitability to replace FM. Thus, in this regard insect meal has emerged as a promising source to replace FM in aquafeed. In the European Union (EU), insect-derived proteins were permitted as pet food (for dogs, cats, birds, or reptiles) and for fur animals (for example, mink). In 2017, clearance was granted to use insects as aquafeed. The EU Member States approved insect processed animal proteins (PAPs) in poultry and pig feed in 2021. Proteins from eight species are permitted to be used as feed, namely, black soldier fly, common housefly, yellow mealworm, lesser mealworm, house cricket, banded cricket, field cricket and, domesticated silkworm. In addition, three insect items as food were permitted in 2021 and 2022: Yellow mealworm (*Tenebrio mollitor* [TM]) in dried, frozen, and powder forms; migrating locust (*Locusta migratoria*) in dry and frozen forms; and house cricket (*Acheta domesticus*) in dried, ground, and frozen forms. Many insect species have been found to be ideal alternative protein sources for human food and animal feed during the past decade due to their multiple benefits [5]. Besides, being highly nutritious [6], they are efficient feed converters [7], and can be easily reared on organic byproducts and agricultural wastes aligned with circular economy strategies [8]. Although insect consumption is still uncommon in western culture, there is a growing openness to embrace insect-based cuisine in some western countries [9]. The nutritional quality of insect meal is comparable to that of conventional FM and their production has a smaller carbon footprint compared to other livestock production [10, 11]. Over the past decade, there has been a significant surge in interest regarding the use of insect meals as a substitute for FM in aquaculture [12]. This trend is evidenced by the exponential growth in scientific research and publications on the use of edible insects as an alternative for FM in aquaculture [3, 13]. It has had a widespread impact and contributed to a large number of highly cited articles. To the best of our knowledge, the present review is the first scientometric review approaching trends in insect meal as replacement to FM in aquaculture. Thus, in the proposed review, the potential of insect meal and their current research trends in aquaculture across the globe in the last decade (2013–2022) will be discussed. Specifically, this study focused on the following key aspects: (a) the temporal patterns in publication trends; (b) the influential authors, institutions, journals, and countries involved in the utilization of insect meal as a substitute for FM in aquaculture; and (c) the current research focal points and anticipated future directions.

## 2. Insect Meal in Aquaculture

Insects are the most diverse group of animals in nature and are a natural food source for carnivorous fishes in their natural habitat due to their higher protein requirements [3, 10]. Humans especially in southeast Asian countries have been using insects as food source for more than 1000 years. Although first use of insects as feeding source for fish was reported in 1980s, the major breakthroughs and research activities started only in the 2010s peaking in the last decade (2013–2022). That's why insect meal has emerged as a popular substitute for FM, where their nutritional profile is quite comparable to the FM. Insect meal contains protein within the range of 50%–82%, which is similar to the FM [14]. Unlike plant ingredients, insect meals are rich in unknown growth factors such as taurine and hydroxyproline in a level comparable to FM [4, 11]. Although many insects have been tried and tested as potential feed source, the major ones include black soldier fly (*Hermetia illucens*), yellow mealworm (TM), silkworms (*Bombyx mori*), house cricket (*A. domesticus*), superworm (*Zophobas morio*), housefly maggot (*Musca domestica*), and mendi termites (*Macrotermes subhyalinus*) are majorly used across the globe due to their higher protein content. The nutritional and amino acid composition of majorly used insect meals are shown in Table 1 (Sources [15–23]) and Table 2 (Sources [15–18, 24–27]).

### 2.1. Black Soldier Fly (*H. illucens*)

*H. illucens* is a common widespread fly belonging to the family Stratiomyidae of order Diptera. Although this species is native to the neotropics, it has recently expanded to all continents, practically becoming cosmopolitan. Pupae and prepupae are consumed by poultry, fish, pigs, lizards, turtles, and even dogs. This insect is one of only a few insect species approved for use as aquaculture feed in the EU. Black soldier flies are at their nutritional peak during the pupal stage. They can be kept at ambient temperature for several weeks and have the longest shelf life at 10–16°C (50–60 °F). Hence, black soldier fly larvae (BSFL) has become a popular choice for replacing FM due to its potential to convert wastes into high quality insect protein [28]. Many studies have been conducted to replace FM using BSFL meal in many species such as *Salmo salar* [29–32], *Oreochromis niloticus* [33–36], *Litopenaeus vannamei* [37–40], *Acipenser bareii* [41], *Lates calcarifer* [42], and *Betta splendens* [43], and it has been found to improve both the growth and immunity [31]. The proximate composition was found to vary based on the substrate reared. The nutritional composition varies as such crude protein (14.6%–62.7%), crude fat (2.8%–38.7%), ash content (2.7%–19.7%). As the lipid content is more the BSFL meal defattening is done to improve the quality of the meal [28].

### 2.2. Yellow Mealworm (TM)

TM are a variety of insects of order coleoptera that grows and reproduces rapidly. They go through four life phases, as do all holometabolic insects: egg, larva, pupa, and adult. Larvae are often 2.5 cm (0.98 in) or larger, although the adults' lengths range from 1.25 to 1.8 cm (0.49–0.71 inch). The yellow mealworm is one of the few promising insect species with the potential for largescale commercial production [44, 45]. Mealworms can be grown in a variety of agricultural and other low-quality organic substrates [46] and used as alternative nutrient sources for livestock, primarily monogastric animals [47]. The nutritional composition varies such as crude protein (47%–63%) and crude lipid (31%–41%) based on the rearing medium. TM has been used to replace FM in many species such as *Micropterus salmoides* [48], *Oncorhynchus mykiss* [49–51], *O. niloticus* [52], *Larimichthys crocea* [53], *L. vannamei* [54], etc.

### 2.3. Superworm (*Z. morio*)

*Z. morio* is a species of darkling beetle, whose larvae are known by the common name superworm, kingworm, or morio worm. The larvae resemble very large mealworms, about 50 to 60 mm (1.7–2.25 inch) long when full size, but unlike mealworms, the ends of their bodies are very dark, almost resembling a black color. They are commonly used in the reptile pet industry as food, along with mealworms (*T. mollitor*). They have been found to improve growth and nutrient utilization in several commercial fish species such as *Sparus aurata* [55], *L. calcarifer* [24], *O. niloticus* [25], *O. mykiss* [56], *and L. vannamei* [57].

### 2.4. Silkworms (*B. mori*)

Sericulture originated in China during the 27th century BC and later spread to other parts of the globe. The global silkworm cocoon market size was valued at USD million in 2022 and will reach USD million in 2028, with a CAGR during 2022–2028. China is the world's single largest producer and the world's leading supplier of silk to global markets. India is the second largest producer in the world. Mulberry, tassar, muga, and eri are the major silk types produced in India. The farmed silkworm (*B. mori*) or mulberry silk is the most common, accounting for 80% of total production. Domesticated silkworm (*B. mori*) pupae are used to produce silk thread from heat-killed pupae fed on mulberry leaves. One cocoon may produce around 800 m of silk-thread, which is a fibroine, a unique elongated molecular thread. The pupae that remain after reeling silk fibre are a waste product of this enterprise and can be used as a feedstuff. This spent silkworm pupae (SWP) is very biodegradable and is typically dumped in the open or used as fertiliser. The use of discarded SWP for feed or the manufacture of useful biological compounds including chitin, protein, oil, and fatty acids (linolenic acid) can be an environmentally acceptable way to reduce the environmental impact of silk production. The nutritional value of dried SWP is comparable to that of FM but at a significantly cheaper cost. Its crude protein concentration ranges from 52% to 72%, whereas de-oiled meal protein content can range from 65% to 80%. SWP protein is rich in essential amino acids such as valine, methionine, and phenylalanine. Thus, SWP meal has been tried in several commercial fish species for replacing high value FM and the results are contradictory. Rahimnejad et al. [58] reported that defatted SWP meal can be used to replace FM upto 75% in the diet of *L. vannamei* with increase in digestibility of nutrients and antioxidant capacity. Sathishkumar, Felix, and Prabu [59] found that bioprocessed SWP meal can replace FM by 66.6% in *O. niloticus*. But Zhang et al. [60] have recently reported that the use of fermented SWP meal beyond 40% reduced growth, feed intake, and body index of largemouth bass (*M. salmoides*).

### 2.5. Housefly Maggot (*M. domestica*)

Housefly maggot (*M. domestica*) meal (HMM) has been demonstrated to be a suitable substitute for traditional protein sources in freshwater aquaculture. Makkar et al. [15] reported that HMM is an economical and ecologically sustainable insect species with high nutritional value (crude protein range between 42 and 60 g/kg). Kolawole and Ugwumba [61] specifically showed how FM could be partially replaced by maggot meal in the diet of African catfish. In 2019, Mustapha and Kolawole [62] found that maggot meal can be used effectively up to 100% to replace FM in the diet of *O. niloticus* fingerlings. Supplementing swamp eels, *Monopterus albus*, with maggot (*M. domestica*) larvae improves their gut flora and fosters growth and immunity [63]. Defatted HMM was also found to entirely replace FM in the diet of African catfish by Fasakin, Balogun, and Ajayi [64] without affecting growth, whereas Akinwole, Duada, and Ogunkunle [65] reported only partial replacement is possible in African catfish (*Clarius gariepinus*). Similarly, Fawole et al. [66] found that HMM in combination with soyabean meal can replace FM completely. On the other hand, Wang et al. [67] found that total FM replacement with HMM in Nile tilapia led to growth depression and subpar feed utilisation.

### 2.6. House Cricket (*A. domesticus*)

*A. domesticus*, also known as the house cricket, is a species of cricket that is probably native to southwestern Asia. However, it expanded throughout the world between 1950 and 2000 as the go-to feeder bug for the pet and research sectors. They can also be maintained as pets, as has been done in China and Japan. While its use in fish feed is still in its infancy, house cricket (*A. domesticus*) meal has been utilized as a source of protein in animal feed [18]. The effect of *A. domesticus* meal on the growth of red hybrid tilapia (*Oreochromis sp*.) in hybrid tilapia has been investigated by Lee et al. [68] and was found that feed containing 60% *A. domesticus* and 40% rice bran yielded good results in terms of growth rate and survival during the feeding trial. A 12-week feeding trial was conducted on perch (*Perca fluviatilis*), in which a mixture of house crickets (*A. domesticus*) was used to replace 25% of the FM. Compared to the control group, fishes fed with *A. domesticus* meal had considerable rise in linoleic fatty acid and a total level of n−6 fatty acids in fish fillets.

### 2.7. Lesser Mealworm or Litter Beetle (*Alphitobius diaperinus*)

*A. diaperinus* (Panzer; coleoptera: Tenebrionidae), also known as the smaller mealworm or litter beetle, is a common insect pest found in commercial chicken production facilities [69]. In poultry farms, it reproduces on the litter floor, where it feeds on organic materials such as dead chickens, shattered eggs, spilled feed, and poultry dung. Due to its recent approval for use as an ingredient in aquafeed and the production of insect meal, *A. diaperinus* has attracted a lot of attention. Because of its high nutritional value, *A. diaperinus*, and especially its larvae, are farmed for a variety of purposes, mostly as feed [17, 70]. However, little information has been gathered from studies looking at the use of *A. diaperinus* as an aquafeed [16]. Chitin levels were shown to be low in pupa and high in adult *A. diaperinus*, according to recent studies [17]. Some studies claim that chitin has a detrimental effect on fish growth performance [71].

### 2.8. Other Potential Insects

Apart from the abovementioned insects, several other insects have also been tried although in a small scale. Sogbesan and Ugwumba [72] tested the potential of termite (*M. subhyalinus*) in the diet of *Heterobranchus longifilis* and found that it can be used up to 50% without any adverse effects. Similarly, the blowfly (*Chrysomya megacephala*) meal was used to replace FM in the diets of red tilapia (*Oreochromis sp*.) by Sing et al. [73]. Results showed blowfly meal improved FCR (feed conversion ratio), SGR (specific growth rate), and survival of the fingerlings. Asadi et al. [74] concluded that locust meal (*L. migratoria*) can be used upto 30% in rainbow trout (*O. mykiss*) with improved growth and immunity.

## 3. Materials and Methods

To identify the development and trends in research, a thorough evaluation of the body of published literature on a given subject is necessary. Metric research can aid in expediting the review procedure in this context by statistically assessing the publication bibliographical data. Scientometrics is the study of quantitative aspects of innovation, science, and technology [75]. Performance analysis and scientific mapping are used to evaluate research publications [76, 77]. Performance analysis is to evaluate the influence and activity of scientific players, such as researchers, nations, organisations, and departments. Citation analysis, cocitation analysis, bibliographic coupling, coword analysis, and coauthorship network analysis are all part of it [78]. The year-by-year increase of publications, cocitation, authorship collaboration, and the geographical distribution of articles, categories of articles, core journals, and significant keywords are the study's analysis criteria. Furthermore, the most influential insect meal research articles have been identified based on citation impacts.

This study found that the scientometric technique and its tools are appropriate for tracking the development of the field and trends in the use of insect meal in aquaculture. Keywords play an important role in a publication [79] and pivotal in scientometric analysis, acting as essential “attributes” for depicting the semantic context of diverse elements like authors, institutions, and citations [80]. These keywords are easy to obtain and have been widely used in previous literatures [14, 16, 24, 81–92]. This review was conducted using the Web of Science Core Collection (WoSCC) database as it is the most reliable and prominent data source comprising the main journals worldwide, and it contains an extensive collection of metadata, including author lists, abstracts, references, citation counts, organizations, journal impact factors, and nations (Table 3) [93, 94]. The time span was set between 2013 and 2022. Languages and document types were set to “all”. The wildcard (*⁣*^*∗*^) was utilized to look for plural form of the word; however, the quotation marks (“”) were used to find exact phrases. Wildcard (*⁣*^*∗*^), quotation marks (“”), and Boolean operator (OR and AND) with a combination of keywords was used to purify the results. All of the original data were obtained from the WoSCC on April 30, 2024. We obtained 354 articles which contained any of these keywords in their title, abstract, or author-keywords. All of which were downloaded as “Plain Text” format file for further scientometric analysis. Article information including author(s), title, source (journal title), language, author-keywords, abstract, publication year, etc. were also exported from the databases. The study used MS Excel to examine the trends in chronological publication and citation increase. For performance measurement, scientific mapping, and network visualisation, VOSviewer [95] and Biblioshiny (Bibliometrix R package) [96] were employed. Tables and charts are used to visually communicate study data.

## 4. Results

A total of 354 publications in the topic of “Insect meal” and “Fish meal replacement” published on the Web of Science (WoS) between 2013 and 2022 were published in 101 different journals (Table 4). Only three studies were produced in other languages: Chinese (1), Hungarian (1), and Russian (1). Given that English is the most common language in the scientific community, the language of the papers was not necessarily related to their nation of origin.

**Figure 1:**
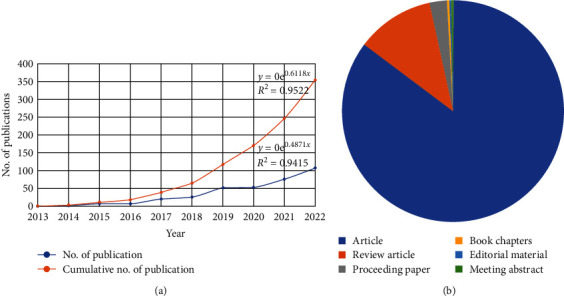
The evolution of the scientific output of the insect meal in aquafeed (A) and types of documents found in the literature (B) in the period (2013–2022).

Figure 1A shows the evolution of the scientific output of the insect meal in aquafeed within the period under study. Out of a total of 354 results, 302 articles, 40 reviews, five proceeding papers, and one book chapter, were categorized (Figure 1B). The 354 publications in the field of “Insect meal” and “aquafeed” on the WoS from 2013 to 2022 were written by 1800 authors. The data collection had 9876 citations in total as of December 2022, with a mean of 36.95 citations per document. Systematic reviews become crucial in this situation to get a comprehensive understanding of the potential of insect meal as an ingredient and alternative protein source for high protein sources such as FM and soyabean meal. Additional research must be done, and it has sparked the scientific community's interest in understanding it's potential in aquafeed industry.

Studies on insect meal in aqua feed have been published in 124 journals. The top 10 journals were chosen based on the quantity of papers submitted.  Table 5 lists the number of citations, publication nations, and impact factor (IF) in 2022. Aquaculture was the journal that published the most publications, with 40 total papers published, or 11.29% of the total, and 2615 citations.

Two prominent measures for analyzing the quantity and quality of articles are the IF and the h-index. The IF was established by the Institute of Scientific Information (ISI) to quantify how frequently a journal's articles are referenced over time, showing the value of a magazine or a series of scientific investigations. The h-index, which considers both number and citations, is another statistic used to assess the publication effect of journals, nations, organizations, or people. The number of papers with at least h citations is specified as the h-index value, whilst other publications (Np-h) have citation counts less than h. The impact of journals and countries on scientific research is assessed in this study using the IF 2022 and the h-index 2022.

**Figure 2:**
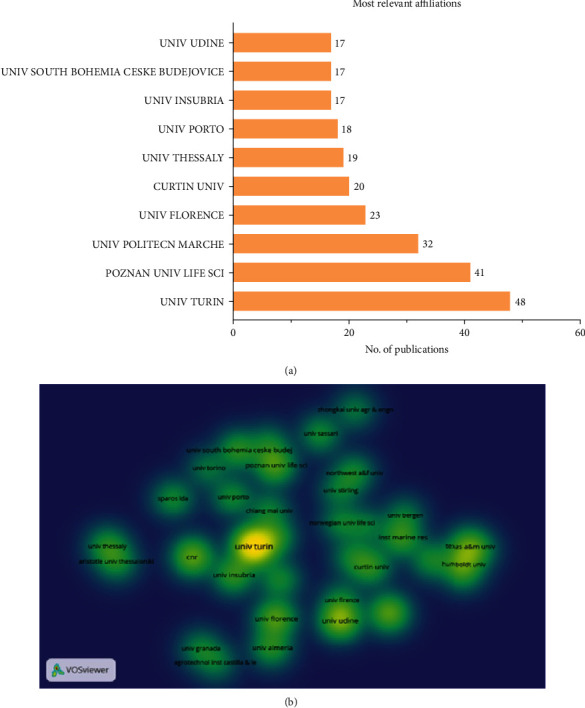
The top 10 most relevant affiliations (A) and density visualization map of organizational linkage (B) based on citations. UNIV, university.

Figure 2A depicts the involvement of several universities in the research. The University of Turin (UNIV TURIN; https://en.unito.it/) was found to be ranked the first, with a total of 48 articles. Poznan University of Life Sciences (POZNAN UNIV LIFE SCI; https://skylark.up.poznan.pl/en/) ranked the second with 41 articles, and Marche Polytechnic University (UNIV POLITECN MARCHE; https://www.univpm.it/Entra/Universita_Politecnica_delle_Marche_Home/L/1) was the third with 32 articles.

A total of 565 organizations participated in the analysis of the organization linkage based on citations, and VOSviewer was used. We selected a minimum number of documents for each organization as five, and 36 organizations met this threshold. University of Turin came out on top with a total of 33 documents with a total of 2444 citations, respectively. Second place went to the University of Udine which had a total of 17 papers and 672 citations, and Consiglio Nazionale delle Ricerche (CNR) occupied the third place with 14 documents (950 citations; Figure 2B).

**Figure 3:**
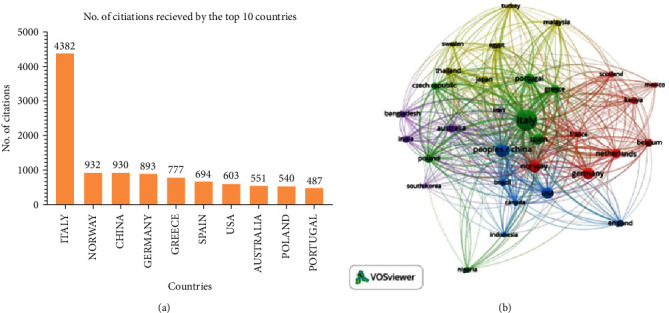
The top 10 most cited countries (A) and countries linkage (B) based on citations received.

Citations to documents illustrate their usefulness to the public and researchers. As illustrated in Figure 3A, the data was evaluated to find the most commonly referenced countries. Italy got the first position with 4382 citations, followed by Norway with 932 citations. China is ranked third overall, with 930 citations. Among a total of 76 nations, participated of which 32 of them had at least five papers. Italy came in first with 91 total documents, followed by China with 41 total documents, as shown in Figure 3B. Network visualisation of top countries revealed that Italy, Spain, Portugal, Greece, Poland, and Czech Republic were grouped under one cluster (green) whereas other European countries such as Germany, Netherlands, Belgium, France, Norway, Scotland, and Kenya were clustered together. In addition, network association was also observed between India, Australia, Iran, South Korea, and Bangladesh.

**Figure 4:**
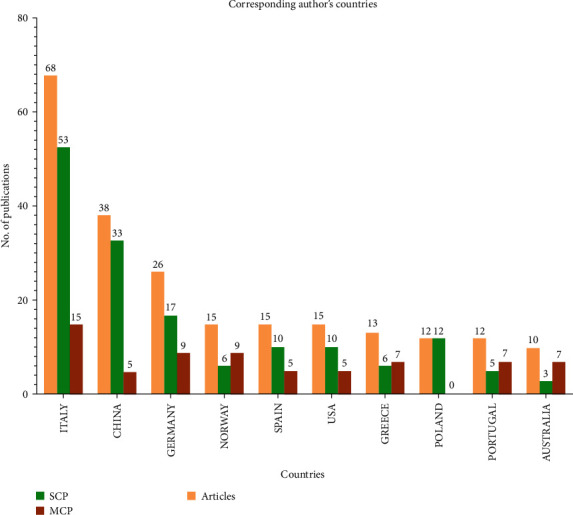
The top 15 nations as presented based on the number of publications. MCP, multiple country publications; SCP, multiple country publications.

Throughout the publishing process, including final proofreading, the corresponding author is the primary point of contact for the manuscript and any associated correspondence. The standards for identifying the relevant author(s) differ from one publisher to the next. For example, although some publishers are liberal when there are multiple related authors on a single document, others are strict when there is only one. The data based on their country of affiliation was analyzed to identify the most relevant corresponding authors. According to the proposed analysis (Figure 4), Italy maintained its dominance with a total of 15 multiple country publications (MCPs) and 53 single country publications (SCPs). China was second with five MCP and 33 SCP.

**Figure 5:**
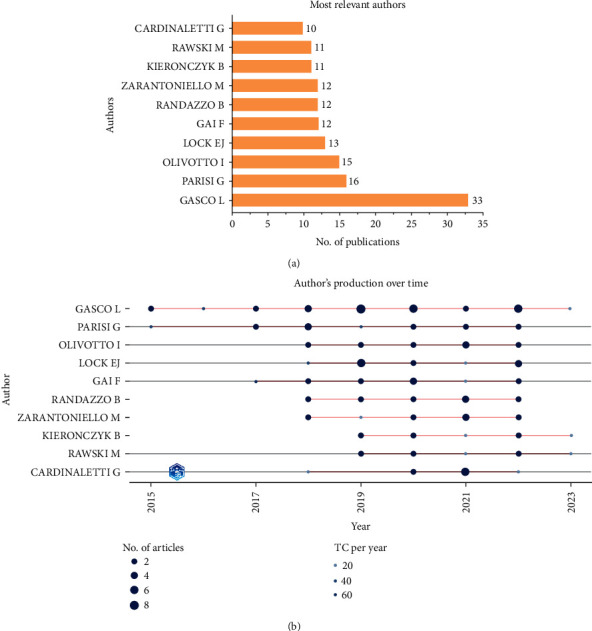
Most relevant authors in insect meal research (A) and their production over time (B) during the study period (2013–2022).

Figure 5A depicts the top 10 most relevant authors in insect meal research during the study period (2013–2022). Laura Gasco of University of Turin published the most amount of research papers (33) followed by Giuliana Parisi of University of Florence who had published 16 papers. Figure 5B shows the authors production over the study period.

A document is typically cited by researchers based on how valuable it is to the public and the scholarly community. A document with more citations has a greater influence. To extract the documents with the broadest scope, we analyzed the data.  Table 6 lists the top 10 most often mentioned documents [14, 15, 26, 29, 97–102]. The most frequently cited publication was a review article titled “State-of-the-art on use of insects as animal feed” which was published in the Animal Feed Science and Technology journal and has a total of 941 citations. Makkar et al. [15] described the potential of insects as a potential feed ingredient for the aquafeed industry.

**Figure 6:**
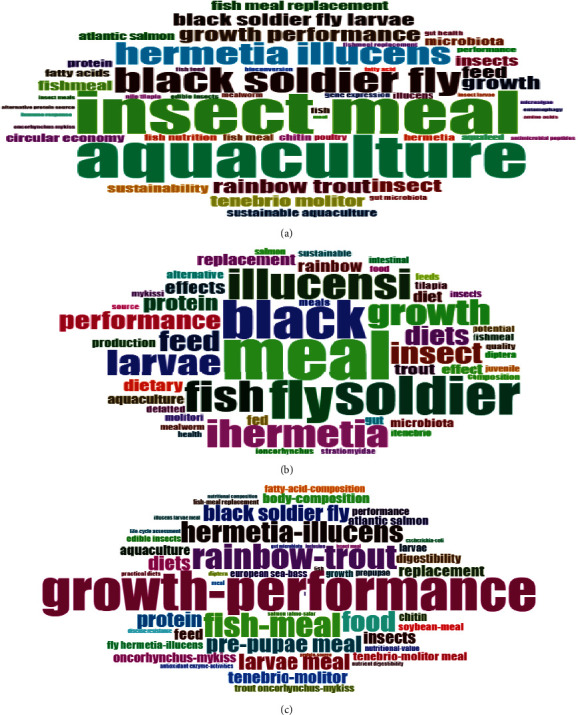
WordCloud generated from the author's keywords (A), titles (B), and keywords plus (C).

The authors analyzed the data to create WordCloud and extracted the words for WordCloud using the author's keywords and titles. “Insect meal” and “Aquaculture” were discovered to be the leading terms recovered from the author's keywords (Figure 6A). However, “meal” and “fly”, among other words, were the leading words retrieved from the titles (Figure 6B). Figure 6C shows similar findings for terms extracted from keywords plus. The terms “growth performance” and “rainbow trout” were tracked down as the leading terms recovered from the keywords plus.

**Figure 7:**
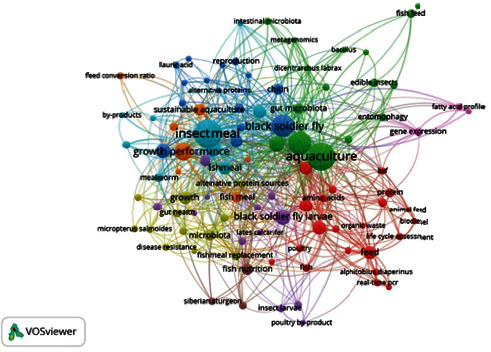
Terms co-occurrence map.

Term co-occurrence map, terms taken from “author's keywords,” 98 terms out of a possible 972 fulfilled the requirement for the term's minimum number of occurrences of three. According to analysis (Figure 7) “insect meal” appeared 71 times, “aquaculture” appeared 64 times, and “*Hermetia illucens*” which was the third most often used phrase, appeared 42 times. Network visualisation of keywords worldwide showed that fish, insects, insect protein, alternative ingredients, alternative protein, aquaculture feed, bioconversion, sustainability, organic waste, and life cycle assessment were observed in a single cluster (red) whereas *H. ilucens*, *M. domestica*, TM, edible insects, probiotics, metagenomics, and bioaccumulation were clustered in one group (green). Circular bioeconomy, FM replacement, waste management, aquafeed, and feed intake formed a separate cluster (blue). The circle's size reflects how often the term appeared as a keyword across various publications. The space between terms shows how often two terms appeared together in the titles, abstracts, or keyword lists of these publications [103, 104].

**Figure 8:**
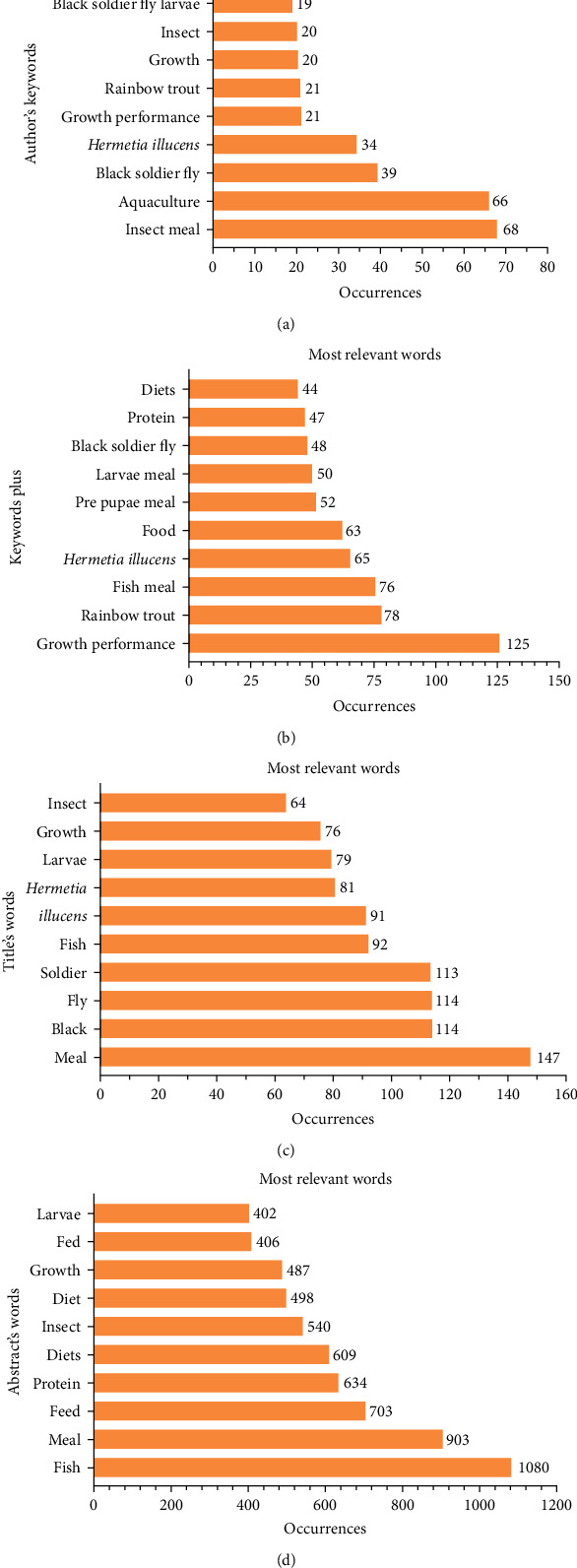
The author's keywords (A), keywords plus (B), titles (C), and abstracts (D) were used to extract the most relevant terms.

The ability to extract the most relevant terms from the data is crucial.  Figure 8 depicts the extraction of the most relevant terms from four distinct groups. The most common phrase in the author's keywords was insect meal, which occurred 68 times, followed by aquaculture, which appeared 66 times (Figure 8A). Growth performance was the most relevant phrase in the keywords plus, appearing 125 times, followed by rainbow trout (78 times) and FM (69 times; Figure 8B). As shown in Figure 8C, meal was the most often common term in the titles, appearing 147 times, followed by fly, which appeared 114 times. The most commonly used phrase in abstracts was fish, which occurred 1080 times, followed by meal, which appeared 903 times (Figure 8D).

**Figure 9:**
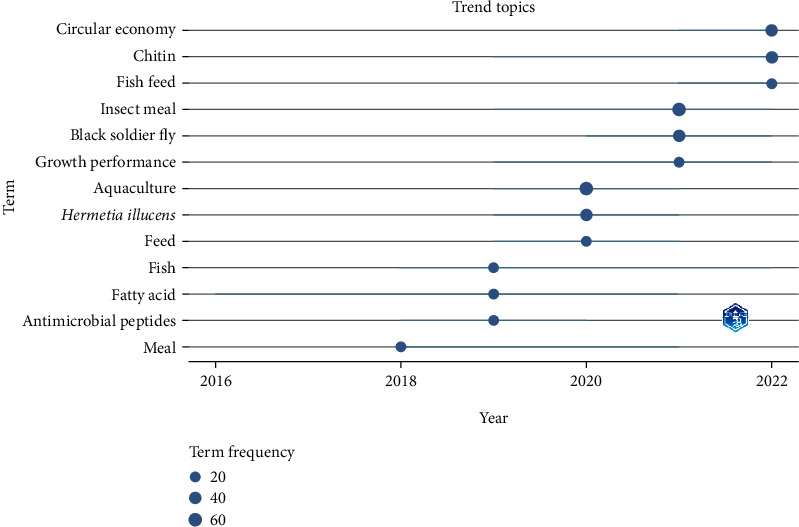
Trending research themes over the years in insect meal.

Figure 9 depicts the trending research themes based on the authors' keywords appearing in the insect meal research. The time period in the Biblioshiny visualisation has been set as 2013–2022, the word minimum frequency as five, and the amount of words each year as five. The figure's circular nodes represent the frequency of appearance of keywords, while the straight line represents the appearance over the year. The chart clearly shows that at first, the research concentrated on the fundamentals, such as the nutrient profile of insect meal, such as amino acids and fatty acids and their quality. Since 2021, the research has been centred on hot subjects like “Circular Economy”. The evolution of the systems indicates that research on insect meal has been ongoing and that it has the potential to occupy a sustainable market economy.

## 5. Discussion

The ongoing expansion of the human population, which is anticipated to reach about 10 billion by 2050, poses a threat to the adequate supply of high-quality food [105]. According to the UN Food and Agriculture Organization (FAO), due to stagnant worldwide catch fisheries productivity, aquaculture has been a crucial component in the steady growth of global fish production for more than a decade [106]. Fed aquaculture, which focuses on delivering farmed fish with formulated feed or processed fish, has been the dominant kind of aquaculture in the last few decades and is certain to grow its share in the future [107]. FM has been the main protein source in formulated feed for many years, and it is still used in contemporary feed formulations. Although the amount of FM in modern feed formulations has drastically decreased compared to earlier times, the demand for FM has not decreased because of the expansion of aquaculture and the related feed industry. The aquafeed sector uses almost 70% of the FM produced globally. Their availability has been greatly impacted by stagnant capture fisheries and shifting climatic circumstances, making their dependence uncertain. As a result, scientists have been searching the world over for substitute sustainable feed sources to take the place of FM. Alternative ingredients, however, must be able to meet a number of criteria, such as wide availability, affordable prices, and specific nutritional qualities, such as high protein content, an appropriate amino acid profile, low carbohydrate and antinutritive factor content, high nutrient digestibility, and better palatability [108, 109]. Insect meal has emerged as a viable replacement for FM due to its capacity to meet the aforementioned requirements, and research is currently being conducted worldwide to determine this. The insect feed market is expected to grow from its current estimate of USD 1.10 billion–1.91 billion by 2028 [4].

Using scientometrics and altmetrics methods, this study examined global research trends on insect meal in aquaculture. A thorough examination of current works can reveal intellectual structure, research hotspots, and developing patterns that can be used to direct future study [77, 78]. It increases the accessibility of available evidence to decision makers, policy planners, and other stakeholders. The analysis provided a detailed summary of the research development as well as the social, intellectual, and conceptual structure of the articles. The examination of the chronological growth of insect meal in aquaculture revealed a great increase in research on insect meal in aquaculture, which is predicted to grow exponentially in the coming years. According to the findings, insect meal research focuses mostly on the nutrient profiling of various insects, their appropriateness for replacing FM, and the sustainability of employing these components in aquaculture. In Europe, the key countries focused on insect meal research are Italy, Norway, Spain, Germany, and Portugal, whereas in Asia, China is the most innovative region with significantly distinct research orientations.

The analysis of the most influential writers and sources was conducted using four performance and citation-based metrics, namely the number of publications, citation, h-index, and g-index. Based on citation counts, this analysis also identified the top 10 most influential articles and most influential journals. Aquaculture (Elsevier) was the leading journal in this research field followed by Animals (MDPI). Similarly, influential organisations and countries were identified using publication, citation, and h-index data. The University of Turin was the leading organisation with the greatest number of articles (48 articles) followed by Poznan University of Life Sciences and University of Turin with 41 articles. Leading organisations from different countries involved in this thematic research shows the growing importance of insect meal as a sustainable feed source in aquafeed industry. Globally and in Europe, Italy was the most cited country in the study period whereas in Asia, China was the leading country followed by India.

Based on citations, the review article “State-of-the-art on use of insects as animal feed” by Makkar et al. [15], in the journal Animal feed science and technology was the most influential article among others. The article empathizes the potential of insect meal in animal feed industry.

The examination of organisation coauthorship (Figure 2B) and country coauthorship (Figure 3B) demonstrates that the collaboration pattern between different authors, organisations, and nations is strong enough in insect meal research.  Figure 8 depicts the year-wise appearance of most prominent author keywords in the insect meal research in aquaculture. Initially, most of the research focused on the nutrient profiling of the insect meal in aquaculture. Of all the insects tried and tested only a few insects such as black soldier fly (*H. illucens*), yellow mealworm (TM), silkworms (*B. mori*), house cricket (*A. domesticus*), superworm (*Z. morio*), and housefly maggot (*M. domestica*) were found to be suitable for replacing FM. The quality of the meal mainly depends upon the life stage of the insects reared and the substrate used for insect rearing [48, 51, 59]. But in the recent years, there has been increasing awareness about the sustainability of usage of insect meal in aquaculture and its impact on health of the animals and consumers [9, 28]. This was reflected in usage of term such as “Circular Economy” which have been prominent in the author's keywords of the globally published research papers (Figure 9).

There are various limitations to the bibliometric analysis. Because WoS continuously updates its database, data received for a certain period may eventually contain information from subsequent publications. The trends revealed by the research are more dependable than the actual data for each location. It is unlikely that the inclusion of a new work will change the most often cited papers, authors, or countries because the majority of the top publishing citations were of sufficient quality. This is evidenced by the fact that some of the most notable trends, such as keywords, countries, and journal sources, are constant in other, more specialised bibliometric analyses. The current study made use of the WoSCC database. In order to determine whether there are any trends for insect meal in aquaculture, future research should evaluate the papers discovered in additional databases. Furthermore, if academics continue to publish in nontraditional places such as open access journals or personal websites, it is possible that certain research will go unreported until it receives a high number of citations [110]. Nonetheless, legislators, stakeholders, or academics may use bibliometric analysis to help design legislation, regulations, or goals for future studies. It applies to articles in all subjects, not just those in information science journals. Instead, it is a feature of single-issue publications and is recognised as a tool in any study, particularly interdisciplinary science.

### 5.1. Key Outcome (Insights of Study)

The field of usage of insect meal in FM replacement is in a stage of rapid development. This article summarizes the recent development in using insect meal in different commercially important aquaculture species and helps in finding the research trend patterns of renowned research institutions globally. Aquaculture has been the fastest animal protein-producing sector in the last decade and is expected to become the major contributor soon. This scientometric analysis helps in mapping the leading journal (Aquaculture), author (Laura Gasco), and institution (University of Turin) in the selected timeframe (2013–2022) of this area of research.

### 5.2. Limitations of This Study

There are some limitations to this research. First of all, only the WoS database was used for the bibliometric analysis, which may lead to a biased picture in results. It is best to add Scopus, PubMed, and other databases for analysis, otherwise many important articles may be excluded. Second, due to the continuous updating of records on WoS database, if the same search were to be performed on a different date, it would probably yield slightly different results. Third, the timeframe of 2013–2022 may not represent a complete picture of the development of usage of insect meal in FM replacement for aquaculture. Fourth, an effort was made to include all possible keywords phrases and research areas relevant to usage of insect meal in FM replacement in aquaculture. However, there could be a possibility of omitting a related keyword or research area.

### 5.3. Future Direction and Policy Suggestions

The article introduces a pioneering scientometric study examining worldwide trends in substituting insect meal for FM in aquaculture research over the last decade. This analysis offers valuable guidance for scholars in exploring new research directions and help them to understand research hot spots and frontiers. Policymakers, stakeholders, or scientists seeking advice on legislation, policies, or future research paths can benefit from the application of this scientometric analysis. The rising costs of aquafeed ingredients, such as meat meal, FM, and soybean meals, which make up 60%–70% of the overall cost of aquaculture production, are impeding the growth of aquaculture. As of now, the EU has approved eight insect species for use in poultry and aquafeeds. The nutritional composition of the insects reared depends mainly on the rearing substrate. Based on these initial findings, improved strains with high potential yield and suitable rearing conditions such as temperature, moisture, and pH can be optimized for mass culture of these insects. Most of the research carried out till now has been lab-oriented small-scale studies. So long-term experiments in large ponds and cages can be done to validate these findings. More insect varieties can also be tried out as insect meal production is more sustainable and has less carbon footprint. Replacing FM with insect meals in aquafeeds necessitates the creation of a legal framework and legislation, along with enhancements in risk assessment procedures. Additionally, research is needed to evaluate how feeding aquaculture species with insect meals affects the safety, quality, and societal acceptance of seafood products. Establishing official standards and regulations for the farming, processing, and safety of insect-based ingredients is crucial for enhancing consumer confidence in the industry. It requires time for the public to appreciate the value of edible insects, whether consumed whole or used as ingredients in aquafeeds. Therefore, significant efforts are also needed in outreach and educational initiatives to promote understanding and acceptance of the insect industry. Since research on insect meal is a multidisciplinary field, this analysis is likely to have an impact on a range of scientific fields and audiences.

## 6. Conclusion

In this study, bibliometric analysis based on 354 relevant papers on “insect meal” and “aquafeeds” from 2013 to 2022 was carried out. Over the last 10 years, publication production has gradually expanded, with substantial international participation. The positive annual growth rate (23.11%) with 36.95 average citations per document shows the growing importance of insect meal in aquafeed feed industry. Although there are many reviews in this thematic research area scientometric studies are very scarce. These preliminary findings will have an impact on research scientists and the aquafeed industry. However, the proposed methodology has some limitations. As VOSviewer requires a uniform data format, the articles were first retrieved from a single database (WoS). To gain more comprehensive data, it can be improved by merging several datasets like Scopus, Google Scholar, and Pubmed. Furthermore, the scientometric analysis in this study is unable to directly present or visualise the future trend, as it is summarised by the authors' expertise, which may lead to it being insufficiently complete. In the future, the publications can be classified into different categories, and the results of scientometric analysis in each category will be submitted to the corresponding expert, where experts from various fields can further advance the research gaps and findings to expand the comprehensiveness.

## Figures and Tables

**Table 1:** Nutritional composition of various insects used in aquafeeds.

Components (%, dry matter)	Protein	Lipid	Crude fibre	Ash
FM	70.6	9.9	1.5	18
Black soldier fly	31.7–47.6 (56–57)	11.8–41.7	23.20	9.85
Yellow mealworm	43.6–53.4 (upto 80)	22.3–30	25.19	4.84
SWP	51.6–70.4	6.2–37.1	2.5–5.8	3.3–10.6
Superworm	33.94–42.81	32.39–44.81	3.2–4.5	2.5
HMM	48.1–50.4 (62.1)	14–37.8%	5.7–6.5	5.3–6.25
House cricket	63.3–71.7 (76.5)	10.4–17.3	7.7	6.3
Banded cricket	59–60.4 (69)	13.3–23.4	7.36	4.84
Lesser mealworm	58–65	13.4–29	7.2	3.6

*Note:* Values shown in bracket () are of defatted meal. *Source:* [15–23].

Abbreviations: FM, fish meal; HMM, housefly maggot meal; SWP, silkworm pupae.

**Table 2:** Amino acid composition (g/16 g nitrogen) of different insect meals compared with FM.

Components (%, dry matter)	FM	Black soldier fly	Yellow mealworm	SWP	Superworm	HMM	House cricket	Banded cricket	Lesser mealworm
Essential amino acids									
Arginine	6.2	5.6	4.8	5.6	5.7	4.6	6.1	5.3	5.6
Histidine	2.4	3	3.4	2.6	3.8	2.4	2.3	3	3
Isoleucine	4.2	5.1	4.6	5.1	6.3	3.2	4.4	4.8	4
Leucine	7.2	7.9	8.6	7.5	8.2	5.4	9.8	8	5.8
Lysine	7.5	6.6	5.4	7	5.8	6.1	5.4	5.9	4.7
Methionine	2.7	2.1	1.5	3.5	0.7	2.2	1.4	1.4	2.3
Phenylalanine	3.9	5.2	4	5.2	5	4.6	3	2.5	3.4
Tyrosine	3.1	6.9	7.4	5.9	6.2	4.7	3	5.2	3.3
Tryptophan	1	0.5	0.6	0.9	0.7	1.5	0.6	0.6	0.8
Valine	4.9	8.2	6	5.5	7.5	4	5.1	6	4
Nonessential amino acids									
Alanine	6.3	7.7	7.3	5.8	3.7	5.8	8.8	9.5	4.6
Aspartic acid	9.1	11	7.5	10.4	7	7.5	7.7	8.8	9.4
Cysteine	0.8	0.1	0.8	1	0.8	0.7	0.8	0.1	1.1
Glutamic acid	12.6	10.9	11.3	13.9	12.5	11.7	10.4	11.7	15.4
Glycine	6.4	5.7	4.9	4.8	2.4	4.2	5.2	5.9	4.8
Proline	4.2	6.6	6.8	5.2	2.5	3.3	5.6	6.2	2.9
Serine	3.9	3.1	7	5	5.4	3.6	4.6	4.9	5

*Source:* [15–18, 24–27].

Abbreviations: FM, fish meal; HMM, housefly maggot meal; SWP, silkworm pupae.

**Table 3:** Literature search strategy.

Criteria	Details
Topic	TS = (“Black Soldier Fly” OR “Black Soldier Fly Larvae” OR “BSF” OR “*H. illucens*” OR “Yellow mealworm*⁣*^*∗*^” OR “Mealworm*⁣*^*∗*^” OR “*Tenebrio molitor*” OR “Superworm*⁣*^*∗*^” OR “*Zophobas morio*” OR “Housefly maggot*⁣*^*∗*^” OR “Magmeal” OR “Common housefly” OR “Common house fly” OR “*M. domestica*” OR “Silkworm*⁣*^*∗*^” OR “Silkworm pupae” OR “*B. mori*” OR “House cricket*⁣*^*∗*^” OR “*Acheta domestica*” OR “Lesser mealworm” OR “*A. diaperinus*” OR “Blue Bottle Fly” OR “*Calliphora vicina*” OR “Banded Cricket*⁣*^*∗*^” OR “*Gryllodes sigillatus*” OR “Jamaican Field Cricket*⁣*^*∗*^” OR “*Gryllus assimilis*” OR “Field Cricket*⁣*^*∗*^” OR “*Gryllus bimaculatus*” OR “Cinerea cockroach” OR “*Nauphoeta cinerea*” OR “American cockroach” OR “*Periplaneta americana*” OR “Grasshopper*⁣*^*∗*^” OR “*Poekilocerus pictus*” OR “*Oxya fuscovittata*” OR “Variegated grasshopper” OR “*Zonocerus variegatus*” OR “Locust” OR “*L. migratoria*” OR “Mendi termite” OR “*Macrotermes subhyalinus*” OR “Rhinoceros beetles” OR “*Oryctes rhinoceros*” OR “Kroto” OR “Weaver ant*⁣*^*∗*^” OR “*Oecophylla smaragdina*” OR “Blowfly larvae” OR “*C. megacephala*” OR “Turkestan cockroach” OR “Blatta lateralis” OR “Maggot Meal*⁣*^*∗*^” OR “Mopane worm*⁣*^*∗*^” OR “Imbrasia belina” OR “Insect meal*⁣*^*∗*^”) AND TS = (Aquaculture OR “Aqua farming” OR “Fish Nutrition” OR “Aquaculture Nutrition” OR “Fish meal replacement” OR “Replacement for fish meal” OR “Fish meal substitute” OR “Fish meal substitution” OR “Fish feed ingredient*⁣*^*∗*^” OR “Insect Meal Inclusion” OR “Soya bean meal replacement”)
Year	2013–2022
Document	All document types
Language	All languages
Database	WoSCC

Abbreviation: WoSCC, Web of Science Core Collection.

**Table 4:** Main information extracted.

Description	Results
Main information about data	
Timespan	2013 : 2022
Sources (journals, books, etc.)	125
Documents	354
Annual growth rate (%)	23.11
Document average age	3.86
Average citations per doc	36.95
References	15,047
Document contents	
Keywords plus (ID)	1018
Author's keywords (DE)	1042
Authors	
Authors	1800
Authors of single-authored docs	4
Authors collaboration	
Single-authored docs	4
Coauthors per doc	7.06
International coauthorships (%)	34.18

**Table 5:** The top 10 most relevant sources ranked by the number of publications.

Element	IF_2022_	h_index	g_index	m_index	TC	NP	PY_start
Animals	3.0	23	36	3.83	1369	38	2019
Aquaculture	4.5	22	40	2.00	2615	40	2014
Journal of insects as food and feed	5.7	14	28	1.40	799	32	2015
Sustainability	11.1	8	15	1.33	368	15	2019
Aquaculture research	10.4	7	9	0.88	87	9	2017
Journal of cleaner production	2.0	7	9	0.88	528	9	2017
Reviews in aquaculture	3.0	7	7	1.17	424	7	2019
Animal feed science and technology	3.9	6	6	0.55	1606	6	2014
Aquaculture nutrition	3.5	6	9	0.67	433	9	2016
Insects	4.6	6	7	0.75	267	7	2017

Abbreviations: NP, number of publications; PY, publication year; TC, total citations.

**Table 6:** Top 10 citations analysis of publications on insect meal in fish feed field.

Article title	Ref.	TC	TC per year	Normalized TC
State-of-the-art on use of insects as animal feed	[15]	941	85.55	2.13
Review on the use of insects in the diet of farmed fish: past and future	[14]	493	49.30	4.45
The future of aquatic protein: implications for protein sources in aquaculture diets	[97]	376	62.67	6.72
The potential of various insect species for use as food for fish	[26]	364	33.09	0.82
Environmental impact of food waste bioconversion by insects: application of life cycle assessment to process using *H. illucens*	[98]	277	34.63	2.59
Evaluation of the suitability of a partially defatted BSFL meal as ingredient for rainbow trout (*O. mykiss* Walbaum) diets	[99]	271	33.88	2.54
Black soldier fly (*H. illucens*) pre-pupae meal as a FM replacement in diets for European seabass (*Dicentrarchus labrax*)	[100]	262	32.75	2.45
BSFL meal can replace FM in diets of sea-water phase Atlantic salmon (*S. salar*)	[29]	258	43.00	4.61
Influence of different growing substrates and processing on the nutrient composition of BSFL destined for animal feed	[101]	224	22.4	2.02
Insect larvae meal as an alternative source of nutrients in the diet of Atlantic salmon (*S. salar*) postsmolt	[102]	220	24.44	3.23

Abbreviations: BSFL, black soldier fly larvae; FM, fish meal; TC, total citations.

## Data Availability

All data are available from the corresponding author upon reasonable request.

## Supporting Information

Supporting Information The supplementary file contains list of abbreviations mentioned within the review.
