# Supplementary material for: Current Status and Global Research Trend Patterns of Insect Meal in Aquaculture From Scientometric Perspective: (2013–2022)
Source: Aquac Nutr. 2024 Oct 22;2024:5466604. doi: 10.1155/2024/5466604 (PMC11521589; doi:10.1155/2024/5466604)
Supplement: Supporting Information — The supplementary file contains list of abbreviations mentioned within the review. [file 5466604.f1.docx]

**Supplementary files**

**Abbreviations:**

**EU- European Union**

**BSFL -Black soldier fly larvae**

**TM *-Tenebrio mollitor***

**FM- Fish Meal**

**SWP -Silkworm pupae**

**HMM- Housefly maggot meal**

**IF- Impact Factor**

MCP -Multiple Country Publications

SCP- Single Country Publications

**FAO -Food and Agriculture Organization**

**WoS- Web of Science**

**WoSCC- Web of Science Core Collection**
